# Factors That Affect Quality of Life among People Living with HIV Attending an Urban Clinic in Uganda: A Cohort Study

**DOI:** 10.1371/journal.pone.0126810

**Published:** 2015-06-03

**Authors:** Doris Mutabazi-Mwesigire, Achilles Katamba, Faith Martin, Janet Seeley, Albert W. Wu

**Affiliations:** 1 Department of Medicine, Makerere University College of Health Sciences, Kampala, Uganda; 2 Department of Psychology, University of Bath, Bath, United Kingdom; 3 Research Unit on AIDS, Medical Research Council/Uganda Virus Research Institute, Entebbe, Uganda; 4 Department of Health Policy and Management, Johns Hopkins Bloomberg School of Public Health, Baltimore, Maryland, United States of America; University of Perugia, ITALY

## Abstract

**Introduction:**

With the availability of antiretroviral therapy (ART) and primary general care for people living with HIV (PLHIV) in resource limited settings, PLHIV are living longer, and HIV has been transformed into a chronic illness. People are diagnosed and started on treatment when they are relatively well. Although ART results in clinical improvement, the ultimate goal of treatment is full physical functioning and general well-being, with a focus on quality of life rather than clinical outcomes. However, there has been little research on the relationship of specific factors to quality of life in PLHIV. The objective of this study was to investigate factors associated with quality of life among PLHIV in Uganda receiving basic care and those on ART.

**Methods:**

We enrolled 1274 patients attending an HIV outpatient clinic into a prospective cohort study. Of these, 640 received ART. All were followed up at 3 and 6 months. Health related quality of life was assessed with the MOS-HIV Health Survey and the Global Person Generated Index (GPGI). Multivariate linear regression and logistic regression with generalized estimating equations were used to examine the relationship of social behavioral and disease factors with Physical Health Summary (PHS) score, Mental Health Summary (MHS) score, and GPGI.

**Results:**

Among PLHIV receiving basic care, PHS was associated with: sex (p=0.045) - females had lower PHS; age in years at enrollment (p=0.0001) - older patients had lower PHS; and depression (p<0.001) - depressed patients had lower PHS. MHS was only associated with opportunistic infection (p=0.01) - presence of an opportunistic infection was associated with lower MHS. For the GPG the associated variables were age (p=0.03) - older patients had lower GPGI; education (p=0.01) – higher education associated with higher GPGI; and depression - patients with depression had a lower GPGI (p<0.001). Among patients on ART, PHS was associated with: study visit (p=0.01), with increase in time there was better PHS, and this also improved with increase in education level (p=0.002). Patients with WHO disease stage 3&4 had a lower PHS compared to patients at stage 1&2 (p=0.006), and depressed patients had lower PHS (p<0.001). MHS improved from baseline to six month study visit (p<0.001), and females had lower MHS compared to males (p=0.01). GPGI was associated with higher income (p=0.04), alcohol use was associated with lower GPGI (p=0.004), and depressed patients had a lower GPGI (p<0.001).

**Conclusion:**

Quality of life improved over time for PLHIV on ART. Regardless of treatment status, PLHIV with depression or low education level and female gender were at risk of having a poor quality of life. Clinicians and policy makers should be aware of these findings, and address them to improve quality of life for PLHIV.

## Introduction

According to Joint United Nations Program on HIV/AIDS, there were 21 million people living with HIV in sub-Saharan Africa in 2012, of whom 32% received antiretroviral therapy (ART) [1]. In the same year, it was reported that Uganda had 1.4 million (1.2–1.6 million) adults living with HIV [2]. By June 2013, 524,603 (37%) adults were on ART [3]. Uganda has had a generalized HIV epidemic for the last 30 years. The HIV prevalence is estimated to be 7.3% [4]. This represents an increase from 6.4% from the 2005 sero-survey [5], which may reflect earlier diagnosis and increased life expectancy of people living with HIV (PLHIV) to a rate similar to the general population [6]. HIV/AIDS has been transformed into a chronic condition, albeit one with no cure, making it important to assess determinants of quality of life (QoL) and, if required, improve the QoL of PLHIV.

Assessment of determinants of QoL is vital to understanding the impact of both HIV and ART on people’s lives [6]. Progress has been made in assessing QoL among PLHIV in developed countries and QoL outcomes have been used in decision making about ARV regimens [7,8,9,10,11]. A recent literature review of determinants of QoL among PLHIV in developed countries reported the following factors to be associated with QoL: gender, age, family situation, education, employment, income, virological and immunological status, staging and time since diagnosis, ART, presence of symptoms, co morbidity, depression and anxiety, social support, neuro psychological status, health care, disclosure, stigma, smoking, alcohol use, drug use, adherence and life style and sexual risk behavior [12]. There is limited evidence of determinants of QoL among HIV/AIDS patients from developing countries. Some studies in developing countries have assessed QoL and its determinants among PLHIV receiving ART [13,14,15,16,17]. Although these studies found improvements in QoL, they were carried out primarily on patients that initiated ART with low CD4 counts (less 200cells/μL), a population in whom it was relatively easy to demonstrate improvement in QoL with a return to health. In the studies that measured QoL among PLHIV on ART specifically, several factors have been identified as relevant to QoL. These factors include depression, age, sex, religion, level of education, CD4 count, marital status, opportunistic infections and socio-economic status were found to be associated with or to affect QoL [8,10,11,13 276,^14^,^15^,^16^]. One wonders whether similar factors are associated with QoL in PLHIV not eligible for ART who have less contact with health workers, or among PLHIV initiating ART at a high CD4 count.

Additionally, other factors have also been reported to be associated with QoL. Wilson and Cleary [18] proposed that behavioral factors determine QoL and these include alcohol use, which has also been associated with risky sexual behavior among Ugandan PLHIV on ART [19]. Poor QoL in people of African origin with HIV has been associated with risky sexual behavior [13]. A study from Cameroon reported poor physical health, frequent unsafe sex, non- disclosure of HIV status and more self reported symptoms among HIV patients not on ART [20]. Understanding the association between these behavioral factors and QoL is crucial since improving QoL may also prevent the spread of HIV. Depression has been reported to be one of the most frequently observed psychiatric disorders among PLHIV [21,22,23] and it has been found to affect QoL negatively [15,24].

The 2010 WHO guidelines that were adopted by Uganda to initiate ART at a CD4 count less than 350 and to give to all pregnant and TB co-infected patients [25] mean that ART is now started when PLHIV are clinically well. The most recent recommendation is to initiate ART at CD4 count less than 500 [26]. The Ugandan government is in the process of adopting this recent recommendation as policy. There are moves towards testing and treating regardless of CD4 count [27], with an aim of reducing risk of onward transmission. Both of these new approaches make QoL a focus of HIV care along with clinical outcomes and mortality, in order to assess patient progress.

To our knowledge, no studies have assessed determinants of QoL among PLHIV not eligible for ART in a limited resource setting among people with a high CD4 count (>350). Monitoring QoL and understanding associated factors can allow targeted interventions that can both improve QoL and reduce risky behaviors. Furthermore, routine assessment of QoL in clinical practice has been shown to improve communication between PLHIV and their care providers [28].

Much as QoL is very important in chronic care, its definition remains contentious. QoL was defined by WHO as “the individuals’ perception of their position in the context of culture and value systems in which they live and in relation to their goals, expectations, standards and concerns” [29]. This definition makes QoL subjective and unique to an individual, his or her culture and environment. The measures of QoL commonly used in clinical practice include items that clinicians consider relevant to the person’s QoL. These more objective measures include symptom severity and level of functioning. Although these measures give the clinician an indication that the PLHIV is doing well or poorly, they ignore the individual’s own perceptions [30]. Measures that define QoL as composed of areas of life defined as important to the individual provide a more subjective assessment, allowing an understanding of both *what* QoL is as well as *how* it is for the individual [31]. Measuring both predefined areas of health related QoL and idiographic global QoL is needed to provide an understanding of the person’s perceived well-being [31].

We undertook this study to identify factors associated with QoL in a cohort PLHIV receiving ART, and PLHIV receiving basic care only because they were not eligible for ART, at an urban clinic in Uganda. We used both an objective and subjective measure of QoL to more fully capture the factors associated with QoL as perceived by the clinician and the individual.

## Methods

### Study design

This was a prospective cohort study that enrolled PLHIV and followed them for six months. Participants were evaluated at enrollment, three months and six months. Participants were enrolled between April 2011 and June 2012.

### Study participants

We enrolled PLHIV from an HIV/AIDS outpatient facility within the national referral hospital. This clinic had registered 21,344 PLHIV by May 2014 with over 60% on ART. PLHIV were eligible for the study if they were documented to be HIV positive, were 18 years or older, and were eligible for ART; or if they were receiving basic care. PLHIV in the ART arm had to be new to ART. Pregnant and terminally ill PLHIV were excluded. We enrolled 1274 PLHIV and all the patients approached responded to the survey questionnaires. Six hundred and forty were eligible for ART and treatment was initiated on the day of enrollment and 634 people not eligible for ART and receiving basic care. All PLHIV eligible for ART (according to the national guidelines CD4 count <350) on a particular clinic day were assessed for enrollment into the study. And a sample of PLHIV in the basic care group not eligible for ART (according to the national guidelines CD4 count >350) were randomly selected per clinic day as they registered.

### Ethical approval

The study was approved by Makerere University College of Health Sciences Higher Degrees, Research and Ethics Committee, and the Uganda National Council for Science and Technology. All participants provided written, informed consent prior to participation in the study.

### Measures

The Medical Outcomes Study (MOS-HIV) Health Survey is the most widely used health related QoL (HRQoL) measure in PLHIV [30]. It is a condition specific instrument that is capable of discriminating between PLHIV on ART and those who are not on ART [32]. Dimensions on the MOS-HIV scale have also been shown to be able to differentiate between asymptomatic and symptomatic PLHIV [33]. A recent review of the HRQoL measures currently in use in HIV/AIDS clinical trials found the MOS-HIV to be one of the two most suitable HIV targeted HRQoL measures [34]. The other measure was the Functional Assessment of HIV Infection (FAHI) [34]. We decided to use the MOS-HIV because it has been validated in various settings including Uganda, in a Luganda language version, and found to be useful in assessing HRQoL in PLHIV [16,35,36,37]. Among Ugandan HIV patients the reliability of PHS and MHS was 0.79 and 0.85 respectively and all the scales with two or more items had cronbach’s alpha value ≥ 0.79 [38]. In another HIV polpulation in Uganda the MOS-HIV was reported to have Crohnbach’s α coefficients > 0.70 for five out of the eight multiitem scale and factor analysis supported the underlysing physical and mental health dimensions [35].

The MOS-HIV measures HRQoL in eleven domains: health perceptions, bodily pain, physical function, role function, social function, mental function, vitality, health distress, cognitive function, QoL, and health transition. The 11 scale scores range from 0–100 with a higher score meaning better health [39]. In addition to these subscales, a Physical Health Summary score (PHS) and Mental Health Summary score (MHS) were calculated by standardizing the scores in these domains using weighting coefficients [39]. The PHS includes physical function, pain, role function, social function and general health and MHS includes cognitive functioning, QoL, health distress, mental health and vitality [39].

Developed from the health focused Patient Generated Index (PGI) [40], the Global Person Generated Index (GPGI) is an assessment of global QoL, as defined by the participant. It has been validated in some developing countries including Ethiopia, Thailand and Bangladesh and found to be useful, valid and reliable in measuring QoL [41,42]. It was recommended to explore needs assessment and response shift [40]. Participants are asked to nominate five areas of life that they perceive as important to their QoL, rate the importance and indicate satisfaction. A final score ranging from 0–100 is produced, weighted by importance [31]. The GPGI allows individuals to define and to rate their own QoL. This questionnaire has three parts. In part one the individual is asked to name five crucial things of their life. In part two they are asked to rate them on a scale of 0 to 6 where 0 is the worst you could imagine and 6 represents exactly how they would like to be. In part three they are asked to spend 10,000 Ugandan shillings to show which areas of their life were the most important to their QoL. They did not have to spend on all items if they did not wish to; however they had to spend all the money. This score was then transformed to a scale of 0 to 10. The global QoL index for each person was calculated by multiplying the scores in part two and with the weights in part three and given as a percentage global QoL score (0–100). A higher score indicates better global QoL. According to Cummins and colleagues [43], scores of subjective well being range between 60 and 80.

The Centre for Epidemiological Depression Scale (CES-D) is a 20 item self report and assessed depressed mood, feelings of guilt and worthlessness, psychomotor retardation, loss of appetite and sleep disorder. It has a score range from 0–60. The higher scores indicate more symptoms for a depressive disorder. The CES-D has been successfully used in Ugandan settings [21,22,23], not only in PLHIV but also in assessment of health related QoL and depression in rural Uganda [15]. The CES-D was validated in a Ugandan HIV population and all the subscales had moderate to high internal consistency with Cronbach’s alpha values of 0.73 and above [22].

The CAGE questionnaire is a screening tool for alcoholism (CAGE is an acronym of the four questions used to screen for alcoholism: Cut down, Annoyed, Guilty and Eye opener) [44,45]. The score range is 1–4. It has been validated for use in identifying alcoholism [46]. It has also been demonstrated that a score ≥2 had a sensitivity of 95% and specificity of 93% to identify excessive drinking and sensitivity of 91% and specificity of 77% to detect alcoholism [47]. The CAGE questionnaire has been shown to have a cross cultural validity and applicability because it does not focus on specifics of alcohol consumption that vary across cultures [48].

### Independent variables

Participants underwent a standardized interview to collect data on socio-demographic characteristics such as sex, age, religion, income per month, employment status, education level and marital status. We collected data on social behavioral characteristics such as smoking, use of alcohol and source of social support. Participants that reported alcohol use were further assessed using the CAGE questionnaire. We used a cutoff score of ≥ 2 to indicate probable alcohol dependency [44]. Information was also collected on clinical characteristics including WHO HIV stage, opportunistic infection, depression and CD4 counts. Blood was drawn at baseline and at the six-month visit for the CD4+ cell count for all participants. All were screened for depression using the CES-D at baseline and at each of the two visits. We used a commonly used cut off of at least 16 to suggest probable depression [49].

### Dependent variables

Two measures were used to collect information on health related QoL at all the three visits. We used a PLHIV report measure specific for HIV: the MOS-HIV, and the subjective measure the GPGI.

### Statistical analysis

We calculated the Cronbach’s α to estimate the reliability of the GPGI. In general a coefficient ≥ 0.70 indicates satisfactory reliability for group comparisons [50]. In order to understand the underlying structure of the GPGI a factor analysis was used. An exploratory factor analysis was done to summarise and group together correlated variables and confirm the presence of a single component in this survey questionnaire. The data supported factor analysis with a large sample size and high Kaiser Meyer Olkin, a correlation matrix displayed the relationship between individual variables and a Principal Component Analysis (PCA) was used to extract the components and rotated the components with varimax rotation to increase interpretability. A scree plot was done to determine the number of components as well. To identify determinants of QoL among PLHIV on ART and those receiving basic care only, we analysed the two groups separately. Descriptive statistics and frequencies were determined for all socio-demographic, clinical and outcome variables. For comparisons between the two groups we used chi square tests for categorical variables, and student’s t—tests and Wilcoxon’s rank sum tests for continuous variables. For the normally distributed outcome variables, we used univariate linear regression with generalised estimating equations to identify factors associated with PHS and MHS. All variables determined to be significantly associated with PHS and MHS in univariate models at a p<0.20 were considered in the multivariate models. For this study we choose p values less than 0.25 based on the finding from Bursac and others, who reported that p values less than 0.25 may indicate some reasonable association with the outcome [51]. All the variables used in this study have been reported to be associated with QoL but a cut- off p value was used to limit the number of variables to ≤10 in order to have a stable model [52]. Despite the p value the following were considered in the final multivariate models: sex, age and study visit (forced variables) because they have been reported in majority of studies to be associated with QoL. We then added all the variables with a p<0.20. We observed the standard errors for the forced variables and found insignificant difference between the two models; this was the final model without multicollinearity. To further test for collinearity, each of the other variables was dropped one at a time from the model and we observed for changes in the standard errors for the forced variables.

The GPGI score was negatively skewed, with the cut- off tail at a score of 60. We therefore categorised GPGI score of less than 60 as low or 60 and above as a high GPGI score. We used logistic regression with generalised estimating equations to identify demographic, behavioural and clinical characteristics associated with GPGI score at univariate and multivariate analysis. The same process was used to build the final multivariate model as was done for the MHS and PHS. The value p<0.05 was used as a cut off for the determinants of QoL at multivariate analysis. We tested for interaction of study arm with time (study visit) to obtain the within subject interaction that may develop overtime and all the significant variables from the multivariate models to test for interaction with study arm. All statistical analyses were conducted using STATA 12.1, Texas USA and IBM SPSS statistics version 20.

## Results

The total number of participants enrolled in the study was 1274 at baseline, of whom 1159 (91%) were also seen at month 6. The reasons for attrition were transferred care center 33 (29%), dead 8 (7%), lost to follow up 74 (64%). At baseline 640 (50%) were in the ART category, and 902 (71%) were female. The mean age in years was 33.6 (SD 8.3). 425 (33%) of participants had a depression score ≥16 on the CES-D scale. The overall Cronbach’s alpha coefficient for GPGI was 0.7 and the PCA extracted a single component that was also confirmed by the scree plot.

Of the 640 participants in the ART group, 481 (75%) received an Efavirenz based regimen and 159 (25%) received a Nevirapine based regimen. The median CD4 count was 417.5 cells/μl for patients on ART and basic care combined (range 259.0–641.0) at baseline and 499.0 cells/μl (range 377.0–642.0) at 6 months. At baseline the median CD4 count of those on ART was 259 (range 151.5, 317.5) and those not on ART had a median of 641 (range 544.0, 801.0). The majority of participants were categorised as WHO stage one and two 1059 (83%) combined ART and basic care. And 80% and 86% WHO stage 1&2 for ART and basic care patients respectively. About a quarter (n = 345; 27%, 32% on ART and 22% on basic care) of participants reported an opportunistic infection at enrolment. This proportion fell to 19% at the 6 month visit. Of the 352 participants who reported drinking alcohol at baseline, 197 (56%) had a score ≥ 2 on the CAGE scale. Table 1 shows the details of the clinical and social demographic characteristics of the study population at baseline by ART status. There were statistically significant differences between the ART and basic care groups for sex, alcohol consumption, WHO stage, opportunistic infection, and median CD4 cell count.

**Table 1:** Characteristics of PLHIV at the baseline visit. Values are frequencies (percentages), except where stated otherwise.

Characteristic	ART group (n = 640)	Basic care group (n = 634)	P value*
**Sex**			
Male	220 (34)	152 (24)	
Female	420 (66)	482 (76)	<0.001
**Mean (SD) age in years**	33.3 (7.9)	34.0 (8.7)	0.09
**Education level**			
Primary or below	233 (50)	327 (51)	
Secondary	225 (40)	251 (40)	
Apprenticeship	44 (7)	46 (7)	
Tertiary	18 (3)	10 (2)	0.50
**Marital status**			
Currently married	378 (59)	393 (62)	
Divorced/separated	161 (25)	140 (22)	
Single	54 (8)	34 (6)	
Widowed	51 (8)	63 (10)	0.06
**Employment status**			
Employed	523 (82)	508 (80)	
Unemployed	117 (18)	126 (20)	0.47
**Income per month in USD**			
<20	195 (30)	201 (32)	
20–60	173 (27)	191 (30)	
>60	272 (43)	242 (38)	0.26
**Religion**			
Catholic	249 (39)	266 (42)	
Protestant	196 (30)	170 (27)	
Moslem	107 (17)	99 (15)	
Pentecostal	76 (12)	89 (14)	
Others	12 (2)	10 (2)	0.42
**Alcohol consumption**			
Yes	152 (24)	200 (32)	
No	488 (76)	434 (68)	0.002
**WHO stage**			
Stage 1&2	513 (80)	546 (86)	
Stage 3&4	127 (20)	88 (14)	0.004
**Opportunistic Infection**			
Yes	205 (32)	140 (22)	
No	435 (68)	494 (78)	<0.001
**Depression**			
No depression (<16)	413 (65)	436 (69)	
Probable depression (>16)	227 (35)	198 (31)	0.11
**Median (IQR) CD4 count (cells/μl)**	259.5 (151.5,317.5)	641.5 (544.0,801.0)	<0.001

Baseline characteristics of study population by ART status

*The p values for the frequencies were analysed using the chi square test, the student’s t-test was used for the mean age, and Wilcoxon rank test was used to measure the CD4 count median association.

**Fig 1:**
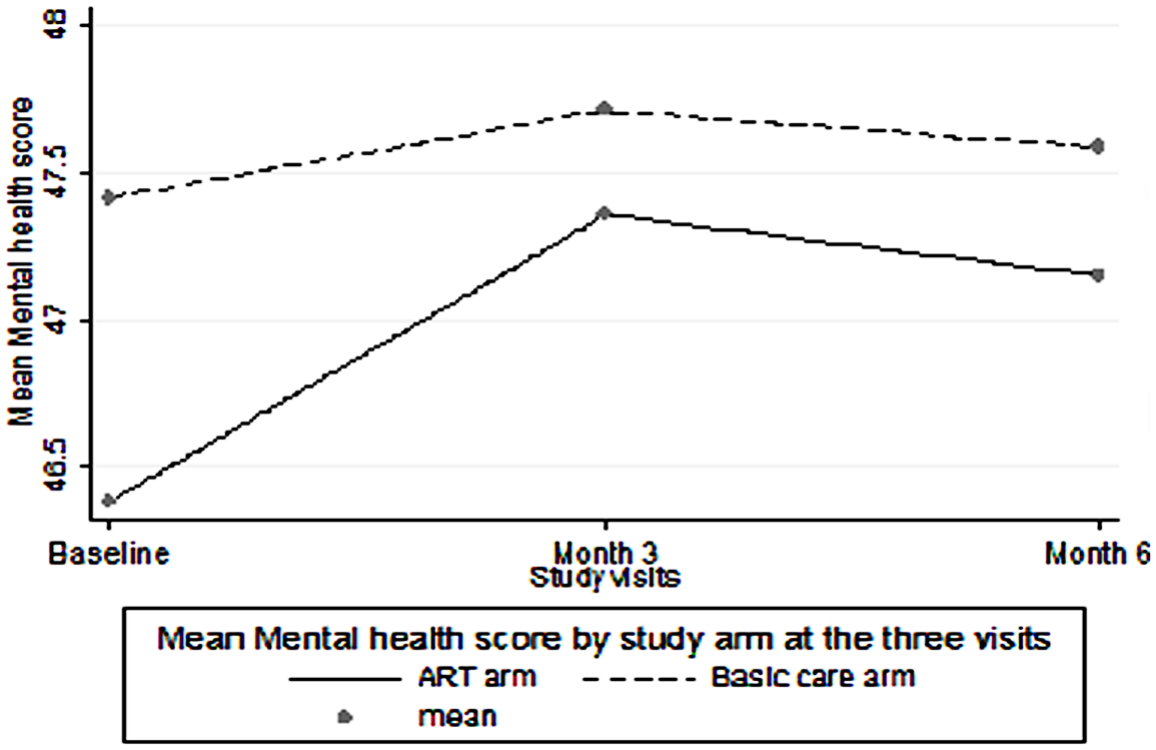
Mean Mental Health Scores over six months. Comparison of Mental Health Score means ART versus basic care among PLHIV over the follow up period.

**Fig 2:**
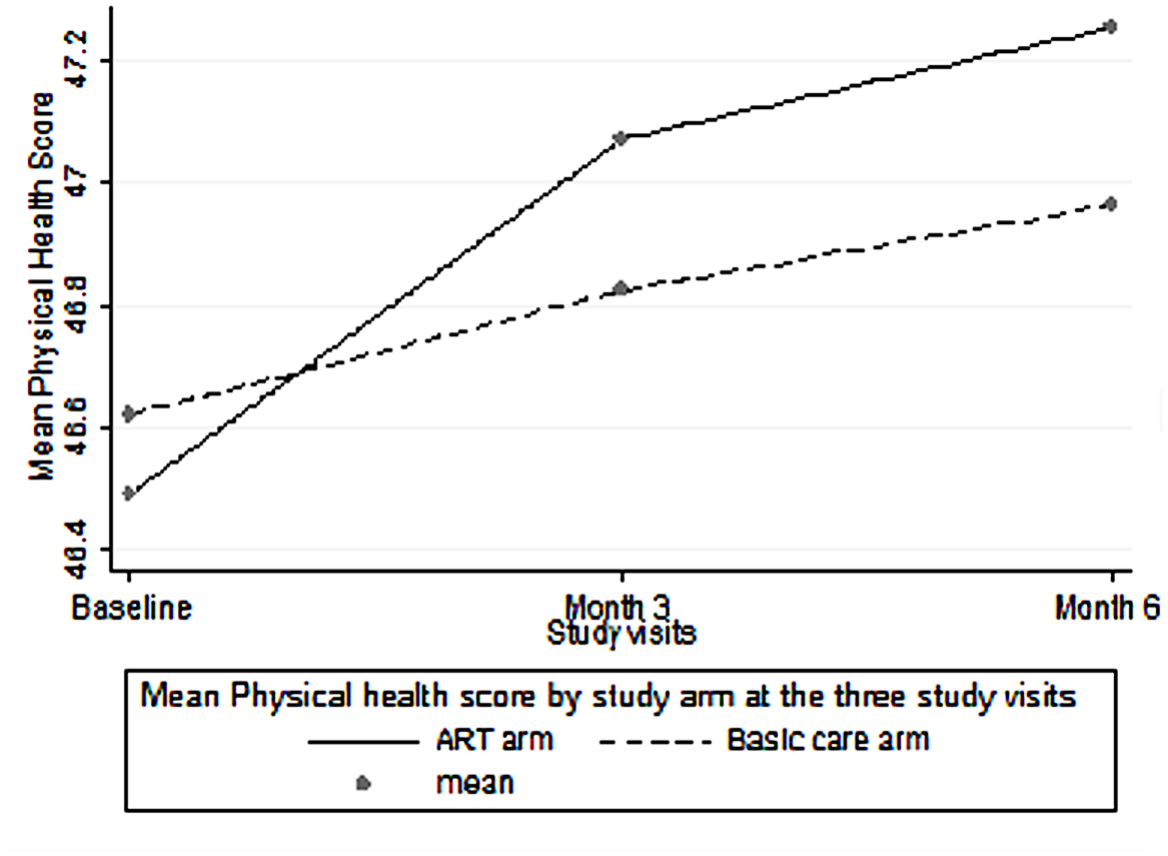
Mean Physical Health Scores over six months. Comparison of Physical Heath Score means ART versus basic care among PLHIV over the follow up period.

**Fig 3:**
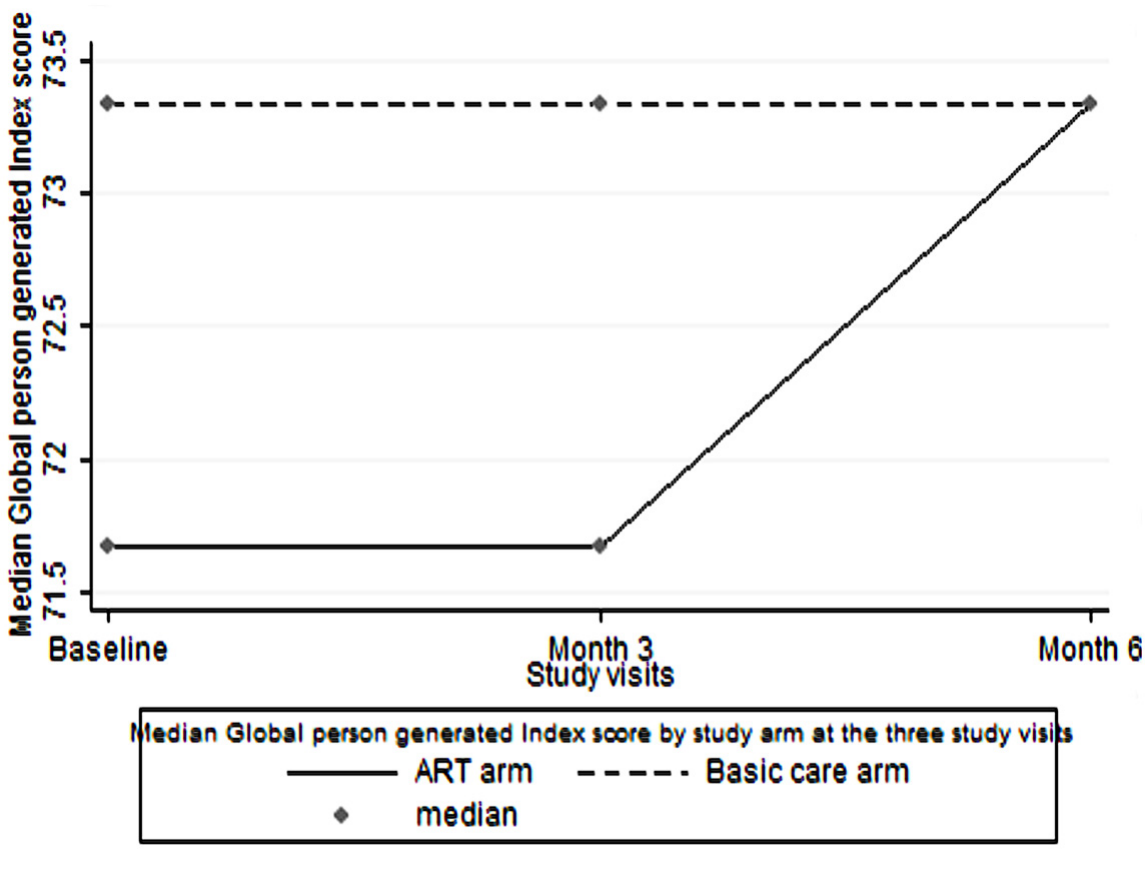
Median Global Person Generated Index score over six months. Comparison of Global Person Generated Index score medians ART versus basic care among PLHIV over the follow up period.

At each of the three visits there was no statistically significant difference between the mean physical health score and the median GPGI score among participants in the ART group compared to those receiving basic care. However, participants receiving basic care had a significantly higher mental health scores compared to participants in the ART group at all the three visits: baseline visit p<0.001, 3 month visit p = 0.04, 6 month visit p = 0.01. (Table 2 and Figs 1–3)

**Table 2:** QoL scores at the three visits. Values are means (SD) and median (range) for Global person generalized Index score.

QoL score	ART	Basic care	P value*
**Physical Health Score**			
**Baseline visit**	**46.5 (4.5)**	**46.6 (3.9)**	**0.59**
**Month 3 visit**	**47.0 (3.9)**	**46.8 (4.0)**	**0.43**
**Month 6 visit**	**47.2 (3.8)**	**47.0 (3.9)**	**0.20**
**Mental Health Score**			
**Baseline visit**	**46.4 (4.5)**	**47.4 (3.8)**	**<0.001**
**Month 3 visit**	**47.3 (3.5)**	**47.7 (3.4)**	**0.04**
**Month 6 visit**	**47.1 (3.4)**	**47.6 (3.3)**	**0.01**
**Global Person Generated Index Score**			
**Baseline visit**	**71.6 (36.7 to 85.0)**	**73.3 (56.7 to 86.7)**	**0.08**
**Month 3 visit**	**71.6 (56.7 to 85.0)**	**75.0 (56.7 to 86.7)**	**0.13**
**Month 6 visit**	**73.3 (58.3 to 85.0)**	**73.3 (60.0 to 85.0)**	**0.78**

Physical Health score, Mental Health score and Global Person Generated Index score at baseline visit, month 3 and month 6

* p value for physical health score and mental health score used the students t test and Wilcoxon rank test was used for the global person generated index score

### Determinants of physical health score over a period of 6 months among PLHIV receiving ART and PLHIV receiving basic care attending the HIV clinic

Factors found to be associated with PHS among PLHIV receiving ART at multivariate analysis were: study visit, education level, WHO stage, and depression. There was an increase in PHS at each study visit compared to the baseline visit (p = 0.01). Specifically, PLHIV on ART had a higher PHS of 0.44 on average at the three month visit compared to the baseline visit (p = 0.02). By month 6 in comparison to baseline visit, the PHS increased by 0.53 (p<0.001). Higher PHS was associated with a higher level of education among PLHIV on ART (p = 0.002). For instance, a University (tertiary level) educated PLHIV on average had a PHS 1.89 higher on average compared to a primary level or no education PLHIV (p = 0.005). PLHIV on ART with probable depression had a 1.75 lower PHS on average compared to PLHIV with no depression at multivariate analysis. A PLHIV graded as WHO stage 3 or 4 had a PHS 0.77 lower than PLHIV in WHO stage 1 or 2. The summary of the factors associated with PHS at univariate and multivariate analysis is shown in Table 3.

**Table 3:** Factors associated with Physical Health Score (PHS) among PLHIV receiving ART and PLHIV receiving basic care using Linear Generalized Estimating Equations.

Characteristic	ART study arm β coefficient (95%CI) (P value)		Basic care study arm β coefficient (95%CI) (P value)	
	Univariate	Multivariate	Univariate	Multivariate
**Study visit**				
Baseline	Ref	**Ref**	Ref	Ref
3 months	0.51 (0.13 to 0.89) (p = 0.009)	**0.44 (0.06 to 0.82) (p = 0.02)**	0.24 (-0.12 to 0.59) (p = 0.20)	0.24 (-0.12 to 0.59) (p = 0.20)
6 months	0.72 (0.33 to 1.10) (p<0.001 overall p = 0.001	**0.53 (0.15 to 0.93) (p = 0.007) overall 0.01 p = 0.59** ^**†**^	0.32 (-0.07 to 0.67) (p = 0.09)	0.32 (-0.06 to 0.69) (p = 0.10)
**Education Level**				
≤Primary	Ref	**Ref**	Ref	Ref
Secondary	0.69 (0.21 to 1.17) (p = 0.005)	**0.44 (-0.03 to 0.90) (p = 0.07)**	0.74 (0.24 to 1.22) (p = 0.003)	0.56 (0.08 to 1.04) (p = 0.02)
Apprenticeship	1.74 (0.81 to 2.66) (p<0.001)	**1.12 (0.32 to 2.10) (p = 0.007)**	0.96 (0.04 to 1.87) (p = 0.04)	0.70 (-0.20 to 1.59) (p = 0.43)
Tertiary	2.31 (0.94 to 3.68) (p = 0.001)	**1.89 (0.5 to 3.21) (p = 0.002) p = 0.44** ^**†**^	1.33 (0.59 to 3.25) (p = 0.17)	0.75 (-1.10 to 2.60) (p = 0.43) overall p = 0.09
**Sex**				
Male	Ref	Ref	Ref	**Ref**
Female	-0.30 (-0.78 to 0.18) (p = 0.22)	0.10 (-0.40 to 0.59) (p = 0.70)	-0.56 (-1.11 to -0.02) (p = 0.04)	**-0.59 (-1.16 to -0.01) (p = 0.045)**
**Age in years**				
<30	Ref	Ref	Ref	**Ref**
30–39	-0.46 (-0.98 to 0.05) (p = 0.08)	-0.44 (-0.94 to 0.06) (p = 0.09)	-0.21 (-0.73 to 0.32) (p = 0.45)	**-0.21 (-0.72 to 0.30) (p = 0.42)**
≥40	-0.56 (-1.17 to 0.05) (p = 0.07)	-0.60 (-1.18 to -0.01) (p = 0.05) overall p = 0.08	-1.16 (-1.77 to -0.55) (p<0.001) overall p = 0.001	**-1.29 (-1.90 to -0.69) (p<0.001) overall p = 0.0001 p = 0.12** ^**†**^
**Income (USD)**				
<20	Ref	Ref	Ref	Ref
20–60	-0.22 (-0.82 to 0.37) (p = 0.46)	-0.24 (-0.81 to 0.34) (p = 0.42)	0.36 (-0.24 to 0.95) (p = 0.24)	0.28 (-0.29 to 0.85) (p = 0.34)
>60	0.77 (0.24 to 1.31) (p = 0.005) overall p = 0.001	0.42 (-0.12 to 0.97) (p = 0.13)	0.80 (0.23 to 1.35) (p = 0.006) overall p = 0.02	0.39 (-0.18 to 0.97) (p = 0.18)
**Religion**				
Christians	Ref	Ref	Ref	Ref
Moslems	-0.41 (-1.02 to 0.20) (p = 0.19)	-0.37 (-0.96 to 0.21) (p = 0.21)	-0.74 (-1.39 to -0.09) (p = 0.03)	-0.67 (-1.30 to -0.04) (p = 0.04)
Others	0.03 (-1.63 to 1.70) (p = 0.97)	0.01 (-1.56 to 1.57) (p = 0.99)	-0.55 (-2.42 to 1.32) (p = 0.56) overall p = 0.07	-0.67 (-2.44 to 1.11) (p = 0.46) overall p = 0.09
**WHO stage**				
1&2	Ref	**Ref**	Ref	Ref
3&4	-1.09 (-1.66 to -0.52) (p<0.001)	**-0.77 (-1.32 to -0.22) (p = 0.006) p = 0.49** ^**†**^	-0.48 (-1.14 to 0.16) (p = 0.14)	-0.48 (-1.14 to 0.16) (p = 0.14)
**Opportunistic infection**				
Yes	Ref	Ref	Ref	Ref
No	0.46 (-0.03 to 0.96) (p = 0.07)	0.20 (-0.27 to 0.68) (p = 0.40)	0.41 (-0.15 to 0.97) (p = 0.15)	0.26 (-0.28 to0.81) (p = 0.35)
**Depression Score**				
<16	Ref	**Ref**	Ref	Ref
≥16	-1.95 (-2.35 to -1.55) (p<0.001)	**-1.75 (-2.16 to -1.35) (p<0.001) p = 0.01** ^**†**^	-1.07 9–1.46 to -0.67) (p<0.001)	**-0.97 (-1.36 to -0.58) (p<0.001) p = 0.01**

Results of univariate and multivariate analysis of Physical Health score among patients on ART and basic care

The significant factors at multivariate analysis are in bold.

^†^p value for interaction term with study arm.

The following factors were significantly associated with PHS among PLHIV receiving basic care: sex, age in years at enrolment and depression. Females had on average a lower PHS by 0.59 compared to the males. There was decrease in PHS associated with increased age (p = 0.0001). PLHIV in the forty years and above category had a lower PHS of 1.29 (p<0.001) on average compared to PLHIV in the thirty years or below age group. PLHIV with probable depression had a 0.97 lower PHS on average compared to PLHIV with no depression. Table 3 shows factors associated with PHS at univariate and multivariate analysis among PLHIV receiving basic care. There was evidence of interaction between study arm and depression score (p = 0.01). There was no evidence of interaction after addition of an interaction term with time (study visit) and study arm in the final model in order to get a within subjects effect (0.59). There was no evidence of interaction between study arm and WHO stage (p = 0.49), age (p = 0.12) or education level (p = 0.44). The other factors significantly associated with PHS (age, education level and WHO stage) showed no evidence of interaction with study arm.

### Determinants of MHS over a period of 6 months among PLHIV receiving ART and PLHIV receiving basic care attending the HIV clinic

Among PLHIV on ART the following factors were associated with MHS: study visit, and sex. Overall, there was an increase in MHS at subsequent study visits compared to the baseline visit (p<0.001). At the three month visit PLHIV had a higher MHS of 0. 94 on average compared to the baseline visit (p<0.001). By the sixth month in comparison to baseline visit, the MHS increased by 0.76. Muslim PLHIV compared to Christian PLHIV had a MHS 0.60 higher on average (p = 0.03). However, overall there was a weak association between religion and mental MHS at a p value = 0.05. The women compared to men on ART had a lower MHS by 0.76 on average.

Presence of an opportunistic infection was the only factor associated with lower MHS among PLHIV receiving basic care. PLHIV with no opportunistic infection had on average a higher MHS by 0.63. Table 4 has details of the univariate and multivariate analysis of factors associated with MHS.

**Table 4:** Factors associated with Mental Health Score among PLHIV receiving ART and PLHIV receiving basic care using Linear Generalised Estimating Equations.

Characteristic	ART study arm β coefficient (95%CI) (p value)		Basic care study arm β coefficient (95%CI) (p value)	
	Univariate	Multivariate	Univariate	Multivariate
**Study visit**				
Baseline	Ref	**Ref**	Ref	Ref
3 months	0.95 (0.59 to 1.31) (p<0.001)	**0.94 (0.58 to 1.31) (p<0.001)**	0.32 (-0.03 to 0.68) (p = 0.07)	0.33 (-0.03 to 0.68) (p = 0.07)
6 months	0.76 (0.39 to 1.12) (p<0.001)	**0.76 (0.40 to 1.13) (p<0.001) overall p<0.001 p = 0.03** ^**†**^	0.20 (-0.16 to 0.56) (p = 0.28)	0.20 (-0.18 to 0.55) (p = 0.31) overall p = 0.19
**Sex**				
Male	Ref	**Ref**	Ref	Ref
Female	-0.87 (-1.30 to -0.43) (p = 0.001)	**-0.76 (-1.22 to -0.30) (p = 0.001) p = 0.04** ^**†**^	-0.13 (-0.58 to 0.33) (p = 0.58)	0.06 (-0.43 to 0.56) (p = 0.81)
**Age in years**				
<30	Ref	Ref	Ref	Ref
30–39	0.46 (0.006 to 0.93) (p = 0.05)	0.36 (-0.11 to 0.84) (p = 0.14)	0.39 (-0.05 to 0.83) (p = 0.08)	0.35 (-0.09 to 0.80) (p = 0.12)
≥40	0.15 (-0.40 to 0.71) (p = 0.59)	0.08 (-0.49 to 0.65) (p = 0.78)	0.29 (-0.22 to 0.81) (p = 0.26) overall p = 0.20	0.22 (-0.32 to0.77) (p = 0.43)
**Religion**				
Christians	Ref	**Ref**	Ref	Ref
Moslems	0.48 (-0.08 to 1.03) (p = 0.09)	**0.60 (0.05 to 1.15) (p = 0.03)**	0.27 (-0.27 to 0.80) (p = 0.33)	0.19 (-0.35 to 0.74) (p = 0.48)
Others	0.70 (-2.21 to 0.81) (p = 0.36) overall p = 0.15	**-0.86 (-2.35 to 0.63) (p = 0.26) overall p = 0.05 p = 0.11** ^**†**^	1.13 (-0.42 to 2.68) (p = 0.15) overall p = 0.24	1.38 (0.16 to 2.92) (p = 0.08) overall p = 0.17
**Marital status**				
Currently married	Ref	Ref	Ref	Ref
Divorced	-0.59 (-1.1 to -0.10) (p = 0.02)	-0.37 (-0.88 to 0.14) (p = 0.16)	0.20 (-0.27 to 0.69) (p = 0.39)	0.18 (-0.30 to 0.67) (p = 0.49)
Single	-0.68 (-1.46 to 0.11) (p = 0.09)	-0.70 (-1.49 to 0.08) (p = 0.08)	-0.31 (-1.18 to 0.55) (p = 0.46)	-0.47 (-1.34 to 0.41) (p = 0.28)
Widowed	-0.73 (-1.51 to 0.05) (p = 0.07) overall p = 0.03	-0.52 (-1.32 to 0.28) (p = 0.20)	-0.03 (-0.68 to 0.63) (p = 0.95)	-0.08 (-0.76 to 0.60) (p = 0.82)
**Social support**				
Yes, family	Ref	Ref	Ref	Ref
Yes, other	-0.19 (-0.80 to 0.41) (p = 0.53)	-0.07 (-0.67 to 0.53) (p = 0.82)	0.28 (-0.21 to 0.77) (p = 0.27)	0.39 (-0.11 to 0.89) (p = 0.13)
None	-1.27 (-2.49 to -0.04) (p = 0.04) overall p = 0.11	-1.25 (-2.47 to -0.03) (p = 0.05) overall p = 0.13	0.70 (-0.21 to 1.61) (p = 0.13) overall p = 0.21	0.63 (-0.28 to 1.53) (p = 0.18)
**Smoking**				
Yes	Ref	Ref	Ref	Ref
No	0.43 (-0.66 to 1.52) (p = 0.44)	0.60 (-0.50 to 1.70) (p = 0.28)	-0.72 (-1.64 to 0.20) (p = 0.12)	-0.74 (-1.69 to 0.20) (p = 0.12)
**WHO stage**				
1&2	Ref	Ref	Ref	Ref
3&4	-0.34 (-0.86 to 0.19) (p = 0.21)	-0.37 (-0.89 to 0.16) (p = 0.17)	-0.38 (-0.94 to 0.18) (p = 0.18)	-0.39 (-0.96 to 0.17) (p = 0.17)
**Opportunistic infection**				
Yes	Ref	Ref	Ref	**Ref**
No	0.30 (-0.15 to 0.75) (p = 0.19)	0.09 (-0.36 to 0.54) (p = 0.69)	0.59 (0.12 to 1.06) (p = 0.01)	**0.63 (0.16 to 1.11) (p = 0.01) p = 0.27** ^**†**^

Results of univariate and multivariate analysis of Mental Health score among patients on ART and basic care

The significant factors at multivariate analysis are in bold.

^†^p value for interaction term with study arm.

### Determinants of high GPGI score over a period of 6 months among PLHIV receiving ART and PLHIV receiving basic care attending the HIV clinic

The following factors were associated with high GPGI score among PLHIV on ART: income per month, alcohol consumption and depression. Overall, the PLHIV with a higher income per month were more likely to have a high GPGI. A person in the category earning more than 60 USD per month had odds of 1.42 (95% CI 0.96 to 2.10) relative to a person in the category earning less than 20USD per month. The odds of high GPGI among PLHIV taking alcohol relative to non-users of alcohol was 1.54 (p = 0.004). PLHIV with probable depression compared to PLHIV without depression had an odds of 0.39 (95% CI 0.31 to 0.49) for a high GPGI.

Age in years at enrolment, education level and depression were the factors associated with GPGI among PLHIV receiving basic care. Overall, there was a reduction in chance of having a high GPGI with increase in age (p = 0.03). The odds of a high GPGI among PLHIV aged 40 years and above, relative to PLHIV aged less than 30 years was 0.64 (95% CI 0.46 to 0.89). There was an increase in chance of having a high GPGI with increase in level of education (p = 0.01). The odds of high GPGI among PLHIV with tertiary education compared to PLHIV with primary school or no education was 10.45 (95% CI 1.60 to 68.20). PLHIV with probable depression relative to PLHIV without depression had an odds of 0.43 (95% CI 0.34 to 0.54) for a high GPGI. Table 5 has details of the univariate and multivariate factors associated with the GPGI.

**Table 5:** Factors associated with high global person Generated Index score among PLHIV receiving ART and to PLHIV receiving basic care using logistic Generalised Estimating Equations.

Characteristic	ART study arm Odds ratio (95%CI) (p value)		Basic care study arm Odds ratio (95%CI) (p value)	
	Univariate	Multivariate	Univariate	Multivariate
**Study visit**				
Baseline	Ref	Ref	Ref	Ref
3 months	1.04 (0.84 to 1.27) (p = 0.73)	1.00 (0.80 to 1.25) (p = 0.99)	1.00 (0.80 to 1.26) (p = 0.96)	1.00 (0.79 to 1.28) (p = 0.96)
6 months	1.38 (1.11 to 1.72) (p = 0.004) overall p = 0.005	1.25 (0.99 to 1.59) (p = 0.06) overall p = 0.07 p = 0.90^†^	1.18 (0.94 to 1.49) (p = 0.16)	1.19 (0.92 to 1.53) (p = 0.17)
**Sex**				
Male	Ref	Ref	Ref	Ref
Female	0.83 (0.64 to 1.07) (p = 0.15)	1.04 (0.78 to 1.40) (p = 0.76)	1.13 (0.84 to 1.52) (p = 0.41)	1.26 (0.92 to 1.73) (p = 0.15)
**Age in years**				
<30	Ref	Ref	Ref	**Ref**
30–39	0.92 (0.70 to 1.20) (p = 0.53)	0.92 (0.69 to 1.22) (p = 0.55)	0.78 (0.58 to 1.05) (p = 0.11)	**0.78 (0.58 to 1.05) (p = 0.10)**
≥40	0.93 (0.67 to 1.30) (p = 0.86)	0.88 (0.62 to 1.25) (p = 0.47)	0.62 (0.45 to 0.87) (p = 0.005) overall p = 0.02	**0.64 (0.46 to 0.89) (p = 0.009) overall p = 0.03 p = 0.29** ^**†**^
**Education level**				
≤Primary	Ref	Ref	Ref	**Ref**
Secondary	1.27 (0.98 to 1.65) (p = 0.007)	1.12 (0.85 to 1.47) (p = 0.41)	1.51 (1.15 to 1.97) (p = 0.003)	**1.35 (1.03 to 1.76) (p = 0.03)**
Apprenticeship	1.17 (0.74 to 1.84) (p = 0.50)	0.91 (0.58 to 1.43) (p = 0.69)	1.75 (1.03 to 2.98) (p = 0.04)	**1.53 (0.91 to 2.59) (p = 0.11)**
Tertiary	3.72 (1.67 to 11.87) (p = 0.03) overall p = 0.05	3.36 (1.15 to 9.87) (p = 0.03) overall p = 0.12	11.85 (1.73 to 80.95) (p = 0.01) overall p = 0.0009	**10.45 (1.60 to 68.20) (p = 0.01) overall p = 0.01 p = 0.44** ^**†**^
**Income USD**				
<20	Ref	**Ref**	Ref	Ref
20–60	1.0 (0.73 to 1.37) (p = 0.99)	**0.97 (0.65 to 1.45) (p = 0.89)**	1.28 (0.93 to 1.77) (p = 0.133)	1.08 (0.76 to 1.54) (p = 0.67)
>60	1.59 (1.20 to 2.12) (p = 0.001) overall p = 0.001	**1.42 (0.96 to 2.10) (p = 0.08) overall p = 0.04 p = 0.45** ^**†**^	1.60 (1.18 to 2.17) (p = 0.003) overall p = 0.01	1.25 (0.88 to 1.77) (p = 0.23)
**Employment status**				
Employed	Ref	Ref	Ref	Ref
Unemployed	0.78 (0.58 to 1.05) (p = 0.001)	0.97 (0.64 to 1.46) (p = 0.88)	0.70 (0.51 to 0.97) (p = 0.03)	0.72 (0.50 to 1.04) (p = 0.08)
**Alcohol consumption**				
Yes	Ref	**Ref**	Ref	Ref
No	1.49 (1.13 to 1.96) (p = 0.005)	**1.54 (1.15 to 2.08) (p = 0.004) p = 0.03** ^**†**^	0.93 (0.71 to 1.22) (p = 0.61)	0.96 (0.72 to 1.26) (p = 0.75)
**Smoking**				
Yes	Ref	Ref	Ref	Ref
No	1.33 (0.73 to 2.42) (p = 0.35)	1.07 (0.55 to 2.08) (p = 0.84)	2.14 (1.19 to 3.84) (p = 0.01)	1.71 (0.96 to 3.06) (p = 0.07)
**Opportunistic Infection**				
Yes	Ref	Ref	Ref	Ref
No	1.33 (1.03 to 1.72) (p = 0.03)	1.19 (0.91 to 1.56) (p = 0.19)	1.06 (0.78 to 1.41) (p = 0.71)	1.03 (0.75 to 1.41) (p = 0.85)
**Depression score**				
<16	Ref	**Ref**	Ref	**Ref**
≥16	0.37 (0.30 to 0.45) (p<0.001)	**0.39 (0.31 to 0.49) (p<0.001) p = 0.40** ^**†**^	0.41 (0.33 t0 0.52) (p<0.001)	**0.43 (0.34 to 0.54) (p<0.001) p = 0.40** ^**†**^
**WHO stage**				
1&2	Ref	Ref	Ref	Ref
3&4	0.76 (0.57 to 1.02) (p = 0.07)	0.85 (0.63 to 1.14) (p = 0.28)	0.86 (0.59 to 1.25) (p = 0.44)	0.99 (0.68 to 1.42) (p = 0.94)

Results of univariate and multivariate analysis of Global Person Generated index score among patients on ART and basic care

The significant factors at multivariate analysis are in bold.

^†^p value for interaction with study arm.

## Discussion

The purpose of this study was to identify factors associated with QoL among PLHIV receiving ART and those not eligible for ART who receive only basic care. Several factors were found to be associated with QoL among PLHIV receiving ART: study visit, education level, WHO stage, depression, sex, income per month and use of alcohol. Among PLHIV on ART, study visit, level of education, WHO stage and depression were determinants of physical health score. There was an increase in PHS from baseline visit to the 6 month visit. PHS also improved with an increase in level of education. The PLHIV on ART and in WHO stage 3&4 had a lower PHS compared to PLHIV in WHO stage 1 &2. PLHIV with probable depression also had a lower PHS. MHS score improved from baseline to the third visit among PLHIV on ART and females had a lower MHS compared to males. Alcohol use, and probable depression were associated with low GPGI score and higher income per month was associated with high GPGI score among PLHIV receiving ART.

PLHIV receiving basic care had on average better mental health scores compared to ART PLHIV on all the three study visits. This might be expected, as they were generally healthier, with higher CD4 counts. Presence of few ART related side effects was associated with “normal” MHS among injection drug users with HIV [53]. It is possible that side effects due to ART initiation in this population may have led to lower MHS. ART may reduce HIV symptoms but also cause side effects [54]. After 4 years of ART, a better MHS was reported compared to before ART initiation [54]. It is also possible that when one stabilizes on treatment the MHS may also improve. Among PLHIV receiving basic care only age, sex, depression, presence of opportunistic infection, and level of education were found to be associated with QoL. Older age, probable depression, and female sex were the determinants of lower PHS. The only factor that was associated with a low MHS among PLHIV receiving basic care was presence of an opportunistic infection. Older age and probable depression were associated with low GPGI among PLHIV receiving basic care, and a higher level of education was associated with a high GPGI score among these PLHIV.

The study population had more women (71%) than men. This is consistent with the clinic population from where participants were drawn, with approximately 70% of the PLHIV being women. Other studies in similar settings have reported a range of 60–75% to be female [13,15,16]. The overall prevalence of depression in this PLHIV population at 33% and is similar to that reported in other PLHIV populations between 30–53% [21,22,23].

Our study found that depression was the only factor negatively associated with PHS and GPGI regardless of ART status. Depression has been reported to be negatively associated with physical health among TB and HIV infected adults in Ethiopia [24]. Studies in the US have also reported depression as a key factor in QoL [55,56]. Depression was associated with lower mental and physical functioning among Spanish PLHIV [10]. Although some studies have reported depression to be associated with only MHS [57,58], we did not analyse for this because the items for MHS were similar to items used to measure depression.

Depression was also reported to affect QoL among Ugandan PLHIV receiving ART at baseline, but was no longer significant after 12 months on ART [15]. This was attributed to the social support and care given while on ART [15]. Women on ART have been reported to have fewer depressive symptoms compared women not receiving ART [59]. It is possible that when PLHIV are initiated on ART, the extra counseling and support given reduce the depression symptoms. Depression in PLHIV has been reported to affect behavior [60], contribute to non-adherence to therapy [61] and to worsen immune functioning [62]. Also, in our study, older age was associated with lower PHS and GPGI. This is consistent with other studies [58,63,64,65]. A mixed population of PLHIV on ART and those not on ART also reported lower QoL scores with the older participants [64]. Our findings may be due to the reduction in biological and physiological b functioning with older age. Older age has been associated with better mental health [66] while in another study older age was associated with decrease in mental health [8]. This association was not significant in our study.

Additionally, we found a higher global QoL and PHS with increasing level of education among patients receiving basic care and ART respectively. This is similar to what has been reported in other studies. PLHIV with post primary education were found to have a better QoL in a study in rural Uganda [15] and in another study at an urban HIV clinic in Uganda [16]. Lower education was found to be associated with lower health related QoL in other settings as well [8,11,59,64]. Lower income and lower education level have been previously found to be associated with lower QoL [64,65]. Income has also been associated with QoL in developed countries [53,58,66]. Higher income per month was also associated with better global QoL in our study. Most likely, socio-economic status improves with higher education. A qualitative study of a subset of PLHIV in our study reported lack of money to negatively affect QoL [67]. It is however notable that PLHIV with a higher education level cope better with HIV. PLHIV with lower education may also face challenges following the treatment recommendations [8]

Some change in QoL was reported in the study from baseline to six months among patients on ART. Improvement in PHS and MHS over the follow up period among PLHIV on ART has also been reported in other studies [15,17,20]. An increase in MHS was also found after ART initiation [68]. It has also been reported that for some PLHIV, QoL may not improve but more instead deteriorate over time [66]. The differences in changes over time in QoL between ART and basic care patients in our study were surprisingly very low and may not have clinical significance. Similar findings with no differences in QoL between ART treated and not treated PLHIV have been reported in other settings [10,53]. Liu and colleagues described the effect of ART on QoL as a balance between reduction of symptoms and side effects [54]. Encountering few side effects from the ART regimens in our study population and the fact that the majority had no HIV symptoms (WHO stage 1& 2) may have contributed to insignificant changes in QoL. It is also possible that six months may be too short a follow up time for other changes that may affect QoL positively or negatively to set in.

We have reported in a qualitative study that women reported being highly stigmatized, lacked disclosure and were solely dependent on men for financial support [67]. Similar to our findings, women in India were reported to have a lower QoL [69]. These conditions may contribute to the lower QoL. Female PLHIV appear to be at risk for having lower health related QoL than men, regardless of ART status. In developing countries, women have also been reported to have lower QoL [10,57]. Some of the possible explanations were that men are expected by society to adapt better [57] and women are more likely to have more guilt feelings compared to men [70]. In contrast women have been reported to have better mental health elsewhere [71].

The presence of HIV related symptoms has been reported to be associated with poor QoL [16,17]. These symptoms are among patients in WHO stage 3&4 of HIV. Similarly, in our study this category of patients was also reported to have a low QoL. Poor PHS has been reported in other studies among HIV patients with AIDS (WHO stage 3&4) developed countries [53,66,72]. These findings confirm that as HIV progresses to another stage the physical health declines.

We found alcohol use to negatively affect QoL, this similar to what has been reported in a previous study [73]. However, no significant association between hazardous drinking and QoL was reported [74]. The few other studies that have assessed alcohol use and QoL reported no significant association with QoL [10,75].

Immunological status (CD4 count) has been reported in several studies among PLHIV receiving ART to be associated with QoL [8,13 275,^15^,^17^,^59^,^64^]. This factor was not included in our models since CD4 count was the main determinant for receiving ART or basic care, and all PLHIV with CD4 <350 received ART in our study.

## Limitations

This study had some limitations. First, we did not assess other psychosocial domains and issues such as sexual function, disclosure and stigma that may affect QoL. The follow up time of six months may be short for PLHIV with a chronic illness. At baseline, one group required ART and the other did not, hence limiting the comparisons. Also, the study was not randomized, making it more difficult to compare PLHIV that received ART to those that received basic care.

## Conclusion

This work contributes to the body of knowledge on QoL among PLHIV receiving ART and basic care in an urban African setting. There was improved QoL with time among PLHIV receiving ART with a CD4 count greater than 350. With the current guidelines to initiate ART early, it is still possible to achieve better QoL while on ART. PLHIV receiving basic care had better mental health compared to PLHIV on ART; this underscores the importance of psycho social support while initiating ART.

QoL among PLHIV in this setting was influenced by several factors simultaneously and probable depression, female gender and a low level of education were determinants of a lower QoL in the absence or presence of ART. Alcohol use and WHO stage 3 & 4 were associated with poor QoL among PLHIV on ART. Older age and presence of opportunistic infections were associated with lower QoL among PLHIV receiving basic care only. A higher was income associated with better QoL among PLHIV receiving ART. These findings suggest that routine assessment for depression and treatment may improve QoL. Timely diagnosis and management of opportunistic infection are vital for PLHIV QoL. Education is crucial in order to improve QoL and this may also improve income. Women, older PLHIV and PLHIV with advanced disease require more support in order to improve their QoL. These factors should be underscored by clinicians and policy makers in the management of PLHIV.

## Recommendations

All PLHIV should be routinely screened for depression and managed appropriately to improve QoL. Psychosocial support to PLHIV on ART should be strengthened improve their mental health. PLHIV with an opportunistic infection may be initiated on ART regardless of CD4 count. All PLHIV should be assessed for alcohol use and appropriate support given. For policy makers, strategies to improve economic wellbeing of PLHIV could be incorporated in routine care. This study also supports the new WHO guidelines to initiate ART at CD4 count >500. This new standard may further optimize PLHIV QoL. We recommend further research to compare objective measures of QoL versus subjective measures with confirmatory factor analysis and structural equation modeling among PLHIV and longer follow up time to assess QoL.

## Supporting Information

S1 Dataset Dataset for quality of life among HIV patients in care. This is the data spread sheet of the 1274 persons used in this study.
(XLS)
